# A crossover study to evaluate the diversion of malaria vectors in a community with incomplete coverage of spatial repellents in the Kilombero Valley, Tanzania

**DOI:** 10.1186/s13071-016-1738-4

**Published:** 2016-08-15

**Authors:** Marta Ferreira Maia, Katharina Kreppel, Edgar Mbeyela, Deogratius Roman, Valeriana Mayagaya, Neil F. Lobo, Amanda Ross, Sarah Jane Moore

**Affiliations:** 1 Swiss Tropical and Public Health Institute, Socinstr. 57, Basel, CH-4002 Switzerland; 2 University of Basel, St. Petersplatz 1, CH-4002 Basel, Switzerland; 3 Ifakara Health Institute, P.O. Box 74, Bagamoyo, Pwani United Republic of Tanzania; 4 University of Glasgow, Institute of Biodiversity Animal Health and Comparative Medicine, Glasgow, G12 8QQ UK; 5 Eck Institute for Global Health, University of Notre Dame, Notre Dame, IN 46556 USA

**Keywords:** Malaria, *Anopheles arabiensis*, *Anopheles funestus*, Spatial repellents, Transfluthrin, Mosquito coils, Diversion, Vector bionomics, Resistance, Tanzania, Kilombero Valley

## Abstract

**Background:**

Malaria elimination is unlikely to occur if vector control efforts focus entirely on transmission occurring indoors without addressing vectors that bite outdoors and outside sleeping hours. Additional control tools such as spatial repellents may provide the personal protection required to fill this gap. However, since repellents do not kill mosquitoes it is unclear if vectors will be diverted from households that use spatial repellents to those that do not.

**Methods:**

A crossover study was performed over 24 weeks in Kilombero, Tanzania. The density of resting and blood-engorged mosquitoes and human blood index (HBI) of malaria vector species per household was measured among 90 households using or not using 0.03 % transfluthrin coils burned outdoors under three coverage scenarios: (i) no coverage (blank coils); (ii) complete coverage of repellent coils; and (iii) incomplete coverage of repellent and blank coils. Mosquitoes were collected three days a week for 24 weeks from the inside and outside of all participating households using mosquito aspirators. Paired indoor and outdoor human landing collections were performed in three random households for six consecutive nights to confirm repellent efficacy of the coils and local vector biting times.

**Results:**

The main vectors were *Anopheles arabiensis* and *Anopheles funestus* (*sensu stricto*), which fed outdoors, outside sleeping hours, on humans as well as animals. *Anopheles arabiensis* landings were reduced by 80 % by the spatial repellent although household densities were not reduced. The HBI for *An. arabiensis* was significantly higher among households without repellents in the incomplete coverage scenario compared to houses in the no coverage scenario (Odds ratio 1.71; 95 % CI: 1.04–2.83; *P* = 0.03). This indicated that *An. arabiensis* mosquitoes seeking a human blood meal were diverted from repellent users to non-users. The repellent coils did not affect *An. funestus* densities or HBI.

**Conclusions:**

Substantial malaria vector activity is occurring outside sleeping hours in the Kilombero valley. Repellent coils provided some protection against local *An. arabiensis* but did not protect against local (and potentially pyrethroid-resistant) *An. funestus*. Pyrethroid-based spatial repellents may offer a degree of personal protection, however the overall public health benefit is doubtful and potentially iniquitous as their use may divert malaria vectors to those who do not use them.

## Background

Insecticide-treated nets (ITNs) protect people from mosquito bites and kill host seeking mosquitoes during sleeping hours and consequently reduce malaria transmission [1]. However, the protective efficacy of ITNs is dependent on a series of factors: the time and place where malaria vectors bite [2], the users’ sleeping hours, correct maintenance of the net, and consistent use of the bed-net [3]. Because the nets prevent mosquitoes from obtaining a blood meal from their human hosts during the late hours of the night, these mosquitoes have been shown to adapt their behavior to host-seeking periods earlier in the evening when people are still active [4–7]. In this case, malaria transmission occurs when people are not yet sleeping under a protective bed net. Rural communities in Tanzania commonly sit outside their homes during and after dusk. In the Kilombero Valley, the majority of the population retires to bed between 9 and 10 pm, until which an estimated 20 % of the total malaria transmission occurs due to early evening biting [8]. Repellents may be a method suitable to provide protection when people are sitting outdoors in the evening.

Repellents are compounds that interfere with the mosquito’s olfactory system preventing them from identifying their hosts and succeeding in taking a blood-meal [9]. These compounds may be applied directly to the skin as a topical formulation; or dispersed into the air creating a mosquito bite free “space” that protects all people within that space. In the latter case they are called spatial repellents. Examples of spatial repellents include mosquito coils, emanators, as well as traditional practices such as burning or smoldering repellent plants [10]. The most common and best-characterized spatial repellent is the mosquito coil. These have been used for centuries in Asia and are traditionally made from a dried paste of pyrethrum powder shaped into a spiral. Currently mosquito coils can be found throughout the world containing volatile pyrethroids such as D-alethrin and transfluthrin. These compounds act as insect neurotoxins and are dispersed into the surrounding air by the smoke of the burning coil. When the mosquito is exposed to the volatilized active ingredient it becomes unable to find a host and feed [11].

Repellents reduce human vector contact but do not kill mosquitoes. Therefore there is a possibility of mosquitoes being redirected (diverted) from repellent users to those individuals who do not use repellents. A previous study conducted in Tanzania investigated the degree of mosquito diversion occurring in villages with incomplete coverage of topical repellents between households that used the topical repellent 15 %-DEET (N,N-Diethyl-meta-toluamide) to households that did not use a repellent [12]. Households not assigned to the repellent group and surrounded by households that did use repellents had three-fold more mosquitoes in and around their dwellings opposed to households in an area where repellents were not used at all. Thus, if complete coverage is not reached topical repellents will divert mosquitoes to households of individuals who do not use them and could potentially increase their exposure to mosquito-borne diseases such as malaria and filariasis. This would lead to health inequity between community members but also would allow pathogens to persist within the community through the vulnerable pockets of population that do not use topical repellents. On the other hand, spatial repellent pyrethroids such as transfluthrin and metofluthrin do not necessarily force the mosquito to move away from the repellent source. Unlike DEET, their main mode of action is neural excitation that results inhibition of host seeking and feeding rather than to repel [11]. However, the host-seeking mosquitoes remain in the environment and may recover from their exposure to the volatile pyrethroid and become ready to host-seek once again. Also, exposure to volatile chemicals outdoors may be insufficient to disarm host-seeking mosquitoes if they simply move away from the repellent source before picking up a sufficient dose. The current study was designed to investigate if diversion of malaria vectors occurs between households that use mosquito coils to those that do not. Health equity implications and the role of spatial repellents as a tool to reduce outdoor residual malaria transmission in the Kilombero Valley are discussed.

## Methods

### Study area

The project was conducted for 24 weeks from December 2012 to June 2013 in Mbingu, which is approximately 40 km west of Ifakara at 8.21°S and 36.24°E, in Kilombero Valley, in south-eastern Tanzania. The site is characterized by typical rural houses surrounded by rice, maize and banana fields close to the Londo River and the slopes of the Udzungwa Mountains. The main mosquito disease vectors are *Anopheles arabiensis*, *An. funestus* (*sensu stricto*) (*s.s.*), *Mansonia africanus*, *Mansonia uniformis*, *Coquillettidia aureus* and *Culex univittatus* [13].

### Study participants

Three villages within the area of Mbingu were selected as study site. A total of 90 households, 30 from each of three villages, Matete, Uwata and Igima, were enrolled in the project. These villages were selected because they had 30 households enabling the crossover design. Geographically, the villages were nearly aligned in a straight line with Igima in the south, Uwata in the north and Matete in between. The distance between Matete and Igima was around 2.5 km and between Matete and Uwata 1 km. Households in the villages were no further than 100 m apart from each other whilst never being directly next to each other (<10 m). All households from these villages agreed to participate in the study; a total 90 households and 381 individuals were enrolled. Each recruited household was given one Olyset® long-lasting insecticide-treated net per sleeping space before the start of the study. Households were numbered and their structural characteristics recorded: wall structure, presence or absence of eaves, roof type, screened or unscreened windows, number of rooms as well as number of occupants and ownership of domestic animals.

### Study design

**Fig. 1:**
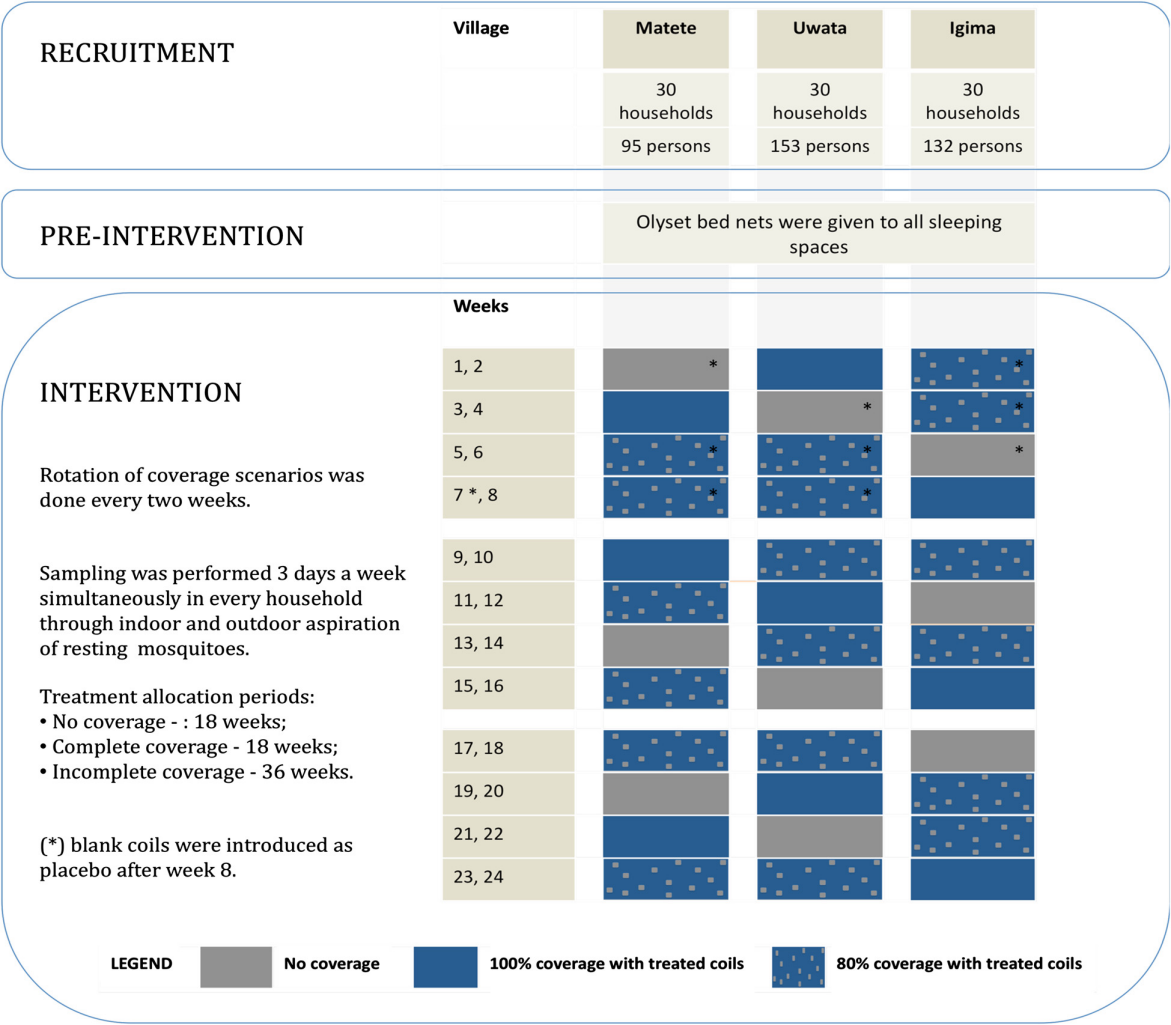
Diagram illustrating the cross-over design used to measure diversion of mosquitoes from households using spatial repellents to households over 24 weeks period

The study was designed to test if mosquitoes are diverted from households that use treated mosquito coils to those that do not. A crossover design was used because of the heterogeneous nature of the data generated from resting mosquito collections from households. This design accounted for differences between households by allowing each household to act as its own control. Three coverage scenarios were rotated between the three villages on a two weekly basis for a period of 24 weeks from December 2012 to May 2013 (Fig. 1). The objective of the study was to measure mosquito densities inside and outside households under three coverage scenarios: (i) Complete coverage; (ii) No coverage; and (iii) Incomplete coverage with 80 % coverage of repellents where 24 households were given treated coils and 6 households were randomly selected using a lottery system and assigned to the repellent non-user group. These households were then removed from the lottery in the following weeks and new houses were randomly selected in order to allow all households to be assigned to the repellent non-user group in the incomplete coverage scenario. The coverage level of 80 % was selected based on a previous study conducted in the same area investigating mosquito diversion caused by topical repellents at 80 % coverage level [12].

Mosquito coils were chosen as the spatial repellent intervention because they are simple to use and well characterized [11]. During the first six weeks households assigned to the no coverage scenario as well as repellent non-users of the incomplete coverage scenario were not given any type of treatment. After week 7 and until the end of the 24 weeks completely identical blank coils were introduced as a placebo. This was done to assess the effect of smoke produced by the blank coils on mosquito densities. Both blank coils and treated coils were produced for this study by SC Johnson and had identical packages, shape, color and lavender smell. Participants were not aware if they were assigned a treated or an untreated coil. Given the nature of a crossover design researchers and the head of the field technicians were not blinded to the intervention. In order to obtain a protective “bubble” effect, household leaders were asked to burn three coils each evening around the space where they sat outdoors in the evenings [14]. Coils were used every day of the week, they were lit at sunset and extinguished once householders retired to bed. Compliance was assessed each morning by inspecting the ashes produced by the mosquito coils during the previous night. Mosquito coils were distributed on a daily basis by the field team and were only sufficient for one night; this was done in order to avoid sharing between households. In addition, the householders were asked what time they had gone to bed the previous evening.

Resting mosquitoes were collected every week on three consecutive weekdays for 24 weeks. Collections were done in all 90 households from all three villages on each experimental day. The team collecting the mosquitoes rotated each between villages to reduce collection bias. Coverage scenarios were assigned to each village on a 2-weekly basis. A total of 72 village/week blocks (3 villages over 24 weeks) were divided between coverage scenarios: (i) Complete coverage: 18 weeks (54 days of resting collections); (ii) No coverage scenario: 18 weeks (54 days of resting collections); and (iii) Incomplete coverage scenario: 36 weeks (108 days of resting collections).

### Resting collections

Resting mosquitoes were collected early morning using Prokopack aspirating devices from under outdoor kitchen thatch roofs as well as indoors [15]. All households were surveyed within a 30-minute window period to avoid differences in resting mosquito densities caused by mosquitoes naturally leaving the households in the mornings. The outdoor kitchens, also known as “kibanda” were selected as previous evaluations had shown them to be a common outdoor resting site for mosquitoes [15]. These structures are a basic thatch roof supported by wooden poles.

Mosquitoes were morphologically identified to genus level and the anophelines were identified to species complex level using morphological keys [16]. A sample of *Anopheles gambiae* (*sensu lato*) (*s.l*.) and *Anopheles funestus* (*s.l*.) was pooled and sent to the IHI laboratory for PCR species identification [17, 18]. A subsample of the PCR confirmed specimens was sent to a laboratory at the University of Notre Dame for quality control. The blood meals of engorged mosquitoes were analyzed using ELISA (enzyme linked immunosorbent assay) to detect the source of blood in engorged mosquitoes: human or animal and allow the calculation of human blood index (HBI).

### Human landing catches

All night indoor-outdoor human landing catches were performed on 6 consecutive nights from dusk till dawn (18:30 to 06:30) during the month of May 2013. The methodology used aimed at reducing all possible risks to collectors and supervisors as described by Gimnig et al. [19]. A total of 6 volunteers were grouped in pairs and each pair was assigned to a randomly selected village household. Each day 3 new households from the study group were randomly selected using a lottery system. A total of 18-paired human landing collections were performed of which 14 were done in households using blank coils and 4 were done in houses using treated coils. This was designed to evaluate the effect of the treatments as well as the biting patterns of the vectors without the spatial repellent. One individual was asked to collect mosquitoes indoors and the other outdoors. After each hour the two individuals swapped positions. All volunteers were males between 18 and 45 years old and recruited under written informed consent. Before starting the mosquito collection each volunteer was seen by a clinical officer who assessed the individual’s general fitness and their malaria status by malaria rapid diagnostic test (mRDT) SD Bioline *Pf* Pan. In order to protect the volunteers from malaria exposure, mefloquine prophylaxis was provided to each individual. In addition, volunteers were asked to wear closed shoes and short trousers and were provided with “bug-jackets” made of untreated netting that completely covered their upper body. Mosquitoes landing on the volunteers bare lower legs were siphoned into a paper cup labeled with time and location. The volunteers were given a hot meal before beginning collections and were allowed 15 minutes break each hour to drink water, tea or use the restroom. The chief field technician supervised the collections and volunteer’s alertness was checked on an hourly basis. The following day a trained technician morphologically identified the mosquitos’ genus or species complex. A subsample of *An. gambiae* (*s.l*.) and *An. funestus* (*s.l*.) was pooled and sent to the IHI laboratory for PCR species identification.

### Statistical analysis

The analyses were carried out in R 2.10.0 [20] using the package *lmer* and *glmm* ADMB [21] and in STATA (version 11), (StataCorp) [22]. The densities of resting and fed mosquitoes were compared between the three coverage scenarios using Generalized Linear Mixed Models [23], in which differences between coverage scenarios were assessed whilst adjusting for village, household and coverage scenario. Mosquito abundance data are typically over-dispersed [24, 25], consequently a negative binomial distribution was used to model the data after assessing their fit to this distribution. Model fit was assessed using a likelihood ratio test and visually by examining the residuals versus fitted plot for deviation from the assumptions of linearity and homoscedasticity. Mosquito densities were compared for the following: (i) household in a no coverage scenario (reference); (ii) household in a complete coverage scenario; (iii) household using repellent coils in an incomplete coverage scenario; and (iv) households not using repellent coils in an incomplete coverage scenario. Coverage scenario was included as a fixed effect together with week to adjust for overall conditions at the time of collection, while village and household were included as random effects. Households assigned to treated coils but that did not comply were re-assigned to “incomplete coverage scenario - repellent non-users” as they did not use a repellent intervention. First, the impact of coverage scenario on resting mosquito and fed mosquito abundance was estimated from the models. We estimated the incidence rate ratios (IRR) and 95 % confidence intervals (95 % CI) of mosquito density per household per night by comparing each coverage scenarios to the coverage scenario where everyone was given a blank coil. Human blood index (HBI) was calculated for each coverage scenario by dividing the total number of mosquitoes fed on humans by the total number of blood-fed mosquitoes. The data for human blood fed as a proportion of blood-fed mosquitoes was analysed with a generalised mixed effects model using a binary distribution with coverage scenario and week included as a fixed effects and village and household as random effects and the odds ratios (OR) and 95 % confidence intervals (95 % CI) were estimated.

Data from the human landing collections was analysed to determine differences between landing rates of mosquitoes when exposed to treated coils opposed to blank coils. A mixed effects Poisson regression with a random intercept to account for overdispersion was carried out in STATA version 11 (Stata Corp) [22]. The presence or absence of the coil was a fixed effect and household and day were random effects. We estimated the IRR of mosquitoes landing on a human per hour when sitting surrounded by three blank coils relative to three transfluthrin 0.03 % coils.

## Results

### Study villages and household participants

Most households of Matete, Uwata and Igima had brick walls with roof made of thatch or metal sheets; eaves were present in virtually all households. The median number of household members was four individuals (Inter-quartile range IQR = 2–6). The median number of rooms was three (IQR = 2–4). Almost every household owned chickens or ducks as domestic animals; goats were common but cattle were found only in one household. Villages were statistically different in terms of roof, wall type and number of rooms per household (Table 1) underlining the importance of the crossover design used. All the villagers cooked outside and ate their meals outdoors before going to bed; the average bedtime was around 21:00.

**Table 1:** Characteristics of the households recruited in Matete, Uwata and Igima and results from statistical tests

Household characteristics		Matete (*n* = 30)	Uwata (*n* = 30)	Igima (*n* = 30)	Chi-square test: *χ* ^2^	*df*	*P*-value
Wall type	Mud	13	3	6			
	Brick	16	27	24			
	Straw	1	0	0	12.1	4	0.017*
Roof type	Thatch	21	14	10			
	Metal Sheets	9	16	20	8.3	2	0.016*
Eaves	Present	27	25	23			
	Absent	3	5	7	1.92	2	0.383
No. of occupants	1–3	20	24	16			
	4–6	9	6	12			
	>7	1	0	2	16.8	14	0.268
No. of rooms	1–3	27	12	19			
	4–6	3	12	9			
	>7	0	6	2	36.4	20	0.014*
Domestic animals	Present	18	20	19			
	Absent	12	10	11	0.29	2	0.866

*P < 0.05

*Abbreviation*: *df*, degrees of freedom

### Malaria vector species composition and biting patterns

Biting patterns were plotted using human landing catch data collected from households using blank coils only. A median number of 19 mosquitoes per household were collected indoors (IQR = 6–40) and a median number of 16 mosquitoes per household were collected outdoors (IQR = 9–27). Sixty-two percent of the collected mosquitoes were *Culex* spp., 24 % *An. arabiensis*, 8 % *An. funestus* and 5 % *Mansonia* spp.

**Fig. 2:**
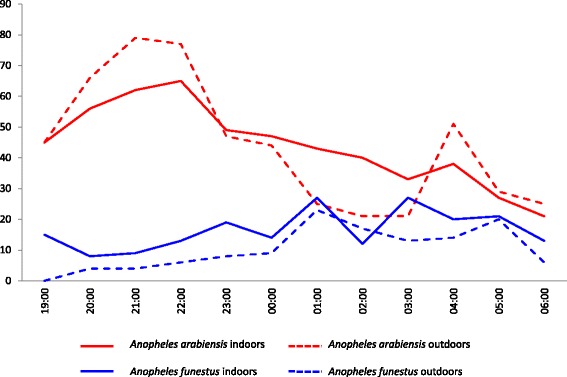
Total number of mosquitoes collected per hour on human landing catch. Biting pattern of *Anopheles arabiensis* and *Anopheles funestus* caught during human landing catches in Mbingu, Kilombero Valley, Tanzania

Results show that *An. arabiensis* were mostly host-seeking during the early evening hours with peak biting time between 20.00 and 22.00 h as well as activity in the early morning (Fig. 2). *Anopheles funestus* preferred biting indoors. Early morning biting was observed for both species. Approximately 23 % of exposure to *An. arabiensis* and 11 % of exposure to *An. funestus* bites occurred outdoors outside sleeping hours.

A total of 1,625 (7.5 % of the total catch) *Anopheles gambiae* (*s.l*.); 1,757 (8.1 % of the total catch) *Anopheles funestus* (*s.l*.); 17,793 (83 % of the total catch) *Culex* spp. (mainly *Cx. quinquefasciatus* and *Cx. univittatus*); 323 (1.4 % of the total catch) *Mansonia africana*/ *M. uniformis*; 13 (0.006 % of the total catch) *Aedes* spp.; and 104 (0.04 % of the total catch) *Coquillettidia aureus/ C. versicolor* were collected from resting catches (Table 2). Human landing catches collected 1,113 (24 %) *An. gambiae* (*s.l*.); 369 (8 %) *An. funestus* (*s.l*.); 2,860 (62.5 %) *Culex* spp.; 211 (4.6 %) *Mansonia* spp.; and 17 (0.4 %) *Coquilettidia* spp. PCR was performed on 1,412 *An. gambiae* (*s.l*.) of which 724 (100 % successful amplifications) amplified as *An. An arabiensis*; 1,393 *Anopheles funestus* (*s.l*.) of which 345 (91 %) amplified as *An. funestus* (*s.s*.); 21 (6 %) as *An. leesoni*; and 11 (3 %) as *An. rivolorum*. Because the amplification rate was low, further PCR amplifications were done for quality assurance at the University of Notre Dame: 163 *An. gambiae* (*s.l*.) were analysed, all amplified, of which 160 (98 %) were *An arabiensis*, 3 (1.5 %) *An. quadriannulatus* and one (0.5 %) *An. gambiae* (*s.s*.); 58 *An. funestus* (*s.l*.) were analysed, 57 (98 %) amplified as *An. funestus* (*s.s*.) and one (2 %) as *An. maculipennis*.

**Table 2:** Effect of 0.03 % transfluthrin coils and blank coils on the number of host-seeking mosquitoes measured by human landing collection

	N^a^	n^b^	GM^c^	95 % CI- GM^d^	IRR^e^	95 % CI- IRR^f^	*P*-value
*Anopheles arabiensis*							
Blank coils	6 (14)	1,056	2.89	2.53–3.3	1	–	–
0.03 % Transfluthrin coils	4 (4)	57	1.41	1.10–1.82	0.22	0.052–0.91	0.037
*Anopheles funestus*							
Blank coils	6 (14)	322	1.85	1.64–2.08	1	–	–
0.03 % Transfluthrin coils	4 (4)	47	1.26	1.12–1.42	0.69	0.21–2.27	0.54
*Culex* spp.							
Blank coils	6 (14)	2243	5.16	4.62–5.76	1	–	–
0.03 % Transfluthrin coils	4 (4)	617	5.22	4.37–6.24	1.27	0.54–2.97	0.59

^a^N, number of nights (number of houses) to each coverage scenario (number of weeks)

^b^n, total number of collected mosquitoes landing on human legs per treatment

^c^GM, geometric mean number of mosquitoes

^d^95 % CI-GM^d^, 95 % confidence interval of GM

^e^IRR, Incidence Rate Ratio

^f^95 %-IRR, 95 % confidence interval of incidence rate ratio

*Abbreviations*: *GM*, geometric mean number of mosquitoes; *IRR*, incidence rate ratio

### Assessment of treatment efficacy

The 0.03 % transfluthrin-treated coils repelled approximately 80 % of the *An. arabiensis* mosquitoes attempting to bite a human outdoors (IRR = 0.22, 95 % CI: 0.052–0.91, *P* = 0.037) (Table 2). In contrast, both *An. funestus* and *Culex* spp. were not significantly repelled by the treated coils (*An. funestus*: IRR = 0.69, 95 % CI: 0.21–2.27, *P* = 0.54; *Culex* spp.: IRR = 1.27, 95 % CI: 0.54–2.97, *P* = 0.59).

The effect of smoke from blank coils did not significantly reduce the number of mosquitoes resting in the households (*An. arabiensis*: IRR = 0.84, 95 % CI: 0.58–1.20, *P* = 0.33; *An. funestus*: IRR = 1.10, 95 % CI: 0.71–1.73, *P* = 0.66; *Culex* spp.: IRR = 0.90, 95 % CI: 0.68– 1.19, *P* = 0.45), but it did elicit a significant reduction in fed mosquitoes of all types (*An. arabiensis*, IRR = 0.53, 95 % CI: 0.33–0.84, *P* = 0.007; *An. funestus*, IRR = 0.56, 95 % CI: 0.34–0.95, *P* = 0.031; *Culex* spp., IRR = 0.16, 95 % CI: 0.10–0.24, *P* < 0.0001) (Table 3).

**Table 3:** The effect of smoke from blank coils on resting and blood feeding mosquitoes collected inside and around households

	N^a^	n^b^	GM^c^	95 % CI- GM^d^	IRR^e^	95 % CI- IRR^f^	*P*-value
Resting *Anopheles arabiensis*							
No coil	12 (4)	129	1.72	1.43–2.06	1	–	–
Blank coils	42 (14)	259	1.44	1.28–1.62	0.84	0.58–1.20	0.328
Blood-fed *Anopheles arabiensis*							
No coil	12 (4)	104	1.62	1.31–2.00	1	–	–
Blank coils	42 (14)	125	1.30	1.14–1.48	0.53	0.33–0.84	0.007
Resting *Anopheles funestus*							
No coil	12 (4)	51	1.11	1.02–1.20	1	–	–
Blank coils	42 (14)	164	1.25	1.16–1.36	1.10	0.71–1.73	0.665
Blood-fed *Anopheles funestus*							
No coil	12 (4)	37	1.10	1.00–1.22	1	–	–
Blank coils	42 (14)	59	1.16	1.10–1.27	0.56	0.34–0.95	0.031
Resting *Culex* spp.							
No coil	12 (4)	1045	3.17	2.83–3.55	1	–	–
Blank coils	42 (14)	3209	3.13	2.91–3.36	0.90	0.68–1.19	0.453
Blood-fed *Culex* spp.							
No coil	12 (4)	322	2.20	1.87–2.58	1	–	–
Blank coils	42 (14)	134	1.35	1.20–1.53	0.16	0.10–0.24	< 0.0001

^a^N, number of nights assigned to each type of no coverage scenario (number of weeks)

^b^n, total number of collected resting mosquitoes

^c^GM, geometric mean number of collected resting mosquitoes

^d^95 % CI-GM^d^, 95 % confidence interval of GM

^e^IRR, Incidence Rate Ratio

^f^95 %-IRR, 95 % confidence interval of incidence rate ratio

### Resting collections and diversion

#### *Anopheles arabiensis*

Diversion of *An. arabiensis* from households using repellent coils to non-users was seen in the incomplete coverage scenario. Human blood index (HBI), for *An. arabiensis* was significantly higher among those households without repellents in the incomplete coverage scenario with HBI increasing from 0.20 to 0.30 (Odds ratio = 1.71, 95 % CI: 1.04–2.83, *P* = 0.03) (Table 4). However, the use of coils had no impact on the probability of finding a resting or a blood-fed *An. arabiensis* in the household (resting: IRR = 0.87, 95 % CI: 0.70–1.09, *P* = 0.21; blood-fed: IRR = 0.91, 95 % CI: 0.68–1.21, *P* = 0.59) (Tables 5 and 6). Nightly *An. arabiensis* densities were highest in households that did not use repellent coils regardless of coverage scenario (households in no coverage scenario IRR = 1; repellent coil non-users in the incomplete coverage scenario IRR = 0.91, *P* = 0.46). The same was observed for blood-engorged females, with highest densities found in households allocated to the no coverage scenario (IRR = 0.86, 95 % CI: 0.62– 1.19, *P* = 0.32) (Table 6).

**Table 4:** Effect of different spatial repellent coverage scenarios on proportion of fed mosquitoes feeding on humans of main malaria vectors

	HBI	OR	95 % CI-OR	*P*-value
*Anopheles arabiensis*				
No coverage	0.20 (46/233)	1	–	–
Complete coverage	0.26 (50/191)	1.21	0.76–1.91	0.43
Incomplete coverage: repellent users	0.23 (78/335)	1.15	0.76–1.75	0.50
Incomplete coverage: repellent non-users	0.30 (40/115)	1.71	1.04–2.83	0.03
*Anopheles funestus*				
No coverage	0.71 (65/91)	1	–	–
Complete coverage	0.66 (123/186)	0.93	0.63–1.37	0.70
Incomplete coverage: repellent users	0.70 (168/240)	0.98	0.67–1.43	0.92
Incomplete coverage: repellent non-users	0.71 (80/112)	1.00	0.65–1.53	1.00
*Culex* spp.				
No coverage	0.30 (35/118)	1	–	–
Complete coverage	0.50 (84/168)	1.26	0.77–2.06	0.36
Incomplete coverage: repellent users	0.47 (242/519)	1.22	0.79–1.89	0.37
Incomplete coverage: repellent non-users	0.50 (89/178)	1.58	0.96–2.62	0.07

*Abbreviations*: HBI, human blood index; OR, odds ratio; 95 % CI-OR- 95 % confidence interval of OR and p-value

**Table 5:** Effect of different spatial repellent coverage scenarios on household mosquito densities

	N^a^	n^b^	IRR^c^	95 % CI-IRR^d^	*P*-value
*Anopheles arabiensis*					
No coverage	54 (18)	427	1	–	–
Complete coverage	54 (18)	319	0.87	0.70–1.09	0.21
Incomplete coverage: repellent users	108 (36)	643	0.91	0.74–1.14	0.42
Incomplete coverage: repellent non-users	108 (36)	233	0.91	0.70–1.17	0.46
*Anopheles funestus*					
No coverage	54 (18)	255	1	–	–
Complete coverage	54 (18)	431	1.44	1.16–1.79	< 0.001
Incomplete coverage: repellent users	108 (36)	710	1.63	1.31–2.02	< 0.001
Incomplete coverage: repellent non-users	108 (36)	350	1.56	1.22–1.98	0.003
*Culex* spp.					
No coverage	54 (18)	4,525	1	–	–
Complete coverage	54 (18)	3,302	0.74	0.66–1.77	< 0.001
Incomplete coverage: repellent users	108 (36)	6,196	0.70	0.63–0.77	< 0.001
Incomplete coverage: repellent non-users	108 (36)	3,626	1.19	1.06–1.34	0.003

^a^N, number of days assigned to each coverage scenario (number of weeks)

^b^n, sum of the total number of mosquitoes collected resting indoors and outdoors per coverage scenario

^c^IRR, Incidence Rate Ratio

^d^95 %-IRR, 95 % confidence interval of incidence rate ratio

**Table 6:** Effect of different spatial repellent coverage scenarios on household densities of blood-fed mosquitoes

	N^a^	n^b^	IRR^c^	95 % CI-IRR^d^	*P*-value
*Anopheles arabiensis*					
No coverage	54 (18)	252	1	–	–
Complete coverage	54 (18)	202	0.91	0.68–1.21	0.59
Incomplete coverage: repellent users	108 (36)	363	0.89	0.67–1.18	0.34
Incomplete coverage: repellent non-users	108 (36)	133	0.86	0.62–1.19	0.32
*Anopheles funestus*					
No coverage	54 (18)	124	1	–	–
Complete coverage	54 (18)	226	1.35	1.01–1.80	0.04
Incomplete coverage: repellent users	108 (36)	289	1.39	1.04–1.86	0.02
Incomplete coverage: repellent non-users	108 (36)	139	1.27	0.91–1.76	0.15
*Culex* spp.					
No coverage	54 (18)	478	1	–	–
Complete coverage	54 (18)	301	0.80	0.60–1.06	0.11
Incomplete coverage: repellent users	108 (36)	634	1.04	0.79–1.37	0.78
Incomplete coverage: repellent non-users	108 (36)	262	1.15	0.84–1.58	0.39

^a^N, number of days assigned to each coverage scenario (number of weeks)

^b^n, sum of the total number of blood fed mosquitoes collected resting indoors and outdoors per coverage scenario

^c^IRR, Incidence Rate Ratio

^d^95 %-IRR, 95 % confidence interval of incidence rate ratio

#### *Anopheles funestus*

No diversion was seen between coil users and non-users with *An. funestus* in the incomplete coverage scenario*.* The HBI was similar in all coverage scenarios ranging between 0.66 and 0.71 and the use of mosquito coils had no effect on the proportion of *An. funestus* fed on humans (Table 4). Unexpectedly, the use of mosquito coils increased the probability of finding *An. funestus* in the household (repellent users in complete coverage scenario: IRR = 1.44, 95 % CI: 1.16–1.79, *P* < 0.001; repellent users in the incomplete coverage scenario: IRR = 1.63, 95 % CI: 1.31–2.02, *P* < 0.001; both compared to no coverage.). Those houses that did not use spatial repellents in the incomplete coverage scenario (where one would expect diversion) also had more *An. funestus* in and around their dwellings compared to when nobody was given a repellent coil (IRR = 1.56, 95 % CI: 1.22–1.98, *P* = 0.003) but this was not different from the densities measured among repellent users and so not indicative of diversion. The number of blood-fed mosquitoes was also 40 % higher among repellent users in both the complete coverage (IRR = 1.35, 95 % CI: 1.01–1.80, *P* = 0.004) and incomplete coverage scenario (IRR = 1.39, 95 % CI: 1.04–1.86, *P* = 0.02). Surprisingly, 30 % of the *An. funestus* (*s.s*.) population had fed on animals (Table 4) indicating that high LLIN use is forcing *An. funestus* to feed on alternate hosts to humans. In addition, very little *An. funestus* activity occurred in the early evening (Fig. 2) so the use of spatial repellents would have a limited impact in preventing man-vector contact with this vector.

#### *Culex* spp.

No diversion was measured with *Culex* species through change in the HBI, with HBI ranging between 0.30 for no intervention and 0.50 for houses with no repellent in the incomplete coverage scenario, and this was not statistically significant (OR = 1.58, 95 % CI: 0.96–2.62, *P* = 0.07) (Table 4). Households not using repellents in the incomplete coverage scenario had 19 % more *Culex* spp. resting in their dwellings (IRR = 1.19, 95 % CI: 1.06–1.34, *P* = 0.003) but the number of blood-fed *Culex* spp. was not significantly different between coverage scenarios. Coils reduced household densities of *Culex* spp*.* (repellent users in the complete coverage scenario: IRR = 0.74, 95 % CI: 0.66–1.77, *P* < 0.001; repellent users in the incomplete coverage scenario: IRR = 0.70, 95 % CI: 0.63–0.77, *P* < 0.001).

### Compliance

There was no withdrawal of study participants throughout the project. Total compliance was measured at a 92 % level and was similar in all coverage scenario groups (no coverage with blank coils: 93 % compliance; complete coverage with treated coils: 91 % compliance; incomplete coverage blank coil users: 90 % compliance; and incomplete coverage treated coil users: 90 % compliance). Reasons given for not complying with lighting the coils included: (i) not being at home that evening; (ii) family had gone to bed earlier than sunset; (iii) very windy weather; or (iv) because some individuals felt unwell for reasons unrelated to the project and the smoke disturbed them, such as “mafua” (Kiswahili word to describe congestion of the nose). Generally the community was pleased to use the mosquito coils and no adverse effects were reported.

## Discussion

Transfluthin mosquito coils reduced mosquito bites from *An. arabiensis* by 80 % among people outdoors although they did not affect densities of *An. arabiensis.* However, households that did not use spatial repellents but were surrounded by households using treated coils had a greater probability of mosquitoes containing a blood meal from a human (Odds ratio 1.71), indicating a slight diversion of bites of *Anopheles arabiensis* to repellent non-users. These findings agree with those of Maia et al. [12] conducted in the same field site where topical repellents diverted mosquitoes to repellent non-using households and increased mosquito densities by over four-fold, although the HBI was not measured in that study and so the results are not directly comparable. The data may also explain to some extent the findings of Wilson et al. [26] demonstrating that mosquito repellents do not reduce malaria at a community scale and that this effect is particularly apparent among communities where compliance with personal protection is lower. Complete coverage of repellent usage at the community level can never realistically be reached and this may lead to diversion of mosquitoes to those who are not protected. In terms of public health benefit it is possible that repellents in general could lead to inequity by increasing the exposure of less informed or poorer individuals to bites of malaria vectors.

The transfluthrin coils did not repel *An. funestus.* This was consistently observed in the data from the human landing collections as well as from the resting collections. Diversion cannot occur if the treatment does not produce a repellent effect that would cause mosquitoes to preferentially feed on repellent non-users. Although the number of *An. funestus* found in households that did not use repellents but were surrounded by repellent users was high, this should not be recognised as diversion. These results indicate that the volatile pyrethroids did not affect the host-seeking behaviour of wild *An. funestus* and are not providing protection against this malaria vector. It is a shortfall of the study that pyrethroid resistance, including susceptibility to transfluthrin was not established. For future studies it is recommended to measure mosquito susceptibility to the repellents used when investigating the effect of volatile pyrethroids on mosquito behaviours, as it has been observed that pyrethroid resistance is a crucial factor that influences the behavioural susceptibility of *Aedes aegypti* mosquitoes to transfluthrin [27].

Households using transfluthrin-treated mosquito coils had fewer indoor and outdoor resting *Culex* spp. Households of repellent non-users in the incomplete coverage scenario had 19 % more *Culex* spp. mosquitoes in their dwellings although the HBI was not significantly different. The variety of *Culex* spp. mosquitoes that occur in this area [13] with potentially different behavioural responses to transfluthrin spatial repellents make it unwise to draw conclusions on diversion for this particular genus as a whole. Unfortunately the study did not identify the culicines to the species level. It would have been interesting to quantify diversion for *Culex quinquefasciatus* in particular, as it is an important disease vector and nuisance agent. It is useful for laboratory and semi-field studies to characterise the behavioural responses of *Cx. quinqefasciatus* to spatial repellents because the nuisance caused by this mosquito may compromise the acceptability of the spatial repellent in the community if mosquito bites are still being perceived.

Results from the human landing collections show that a considerable proportion of exposure to malaria vectors is occurring during the early evening hours. *Anopheles arabiensis* biting densities were highest at 21:00 h, which coincides with the mean bedtime in the area. During recent years there have been consistent reports of vector biting activity shifting towards early evening hours in Kenya [7, 28], Mozambique [29], Zambia [30] as well as Tanzania [31, 32]. Classically malaria vectors in sub-Saharan Africa were mostly host-seeking in the middle of the night [33]. The shift to early evening biting is thought to occur due to high levels of LLIN coverage which has pressured the vector population to modify biting patterns in order to feed on a human host when he or she is available [34]. Data from previous studies in the nearby village of Namwawala, show that since the 1990s there is an increasing trend in outdoor biting and early evening activity of malaria vectors [32]. The village of Mbingu lies approximately 20 km southwest of Namwawala and although retrospective data are not specifically available for Mbingu it is likely that a similar phenomenon has occurred. Also, changes in species composition of the main malaria vectors in Mbingu have taken place. A study conducted in the same villages in 2010 reported collecting 83 % *An. gambiae* (*s.s*.), 17 % *An. arabiensis* and absence of *An. funestus* [12]. Since then *An. gambiae* (*s.s*.) has nearly disappeared and been replaced by *An. arabiensis*. It is also remarkable how *An. funestus* progressed from nearly absent to one of the main vector species of the Kilombero valley within two years despite universal bed-net coverage being in place [11, 35, 36]. Given the classical anthropophilic and endophilic character of *An. funestus* it would have been expected that LLINs reduce the vector densities as happened with *An. gambiae* (*s.s*.). It is unlikely that LLINs alone are the reason why *An. gambiae* (*s.s*.) has been eliminated from the Kilombero Valley, environmental factors such as competition for breeding sites and weather may have also played a role. The present study observed a large proportion of *An. funestus* feeding on animals with HBI ranging between 0.66 and 0.71. Classically *An. funestus* is an almost strictly anthropophilic vector with HBI around 0.95 [37, 38]. Between 1990 and 1992 resting catches were performed just a few kilometres north of Mbingu, hundreds of blood engorged *An. funestus* were collected and virtually all had fed on humans [39]. The present study reports a variation in the host-feeding patterns of *An. funestus* in the Kilombero Valley. Given the selective pressure exerted by LLINs *An. funestus* mosquitoes may have been forced to adapt their host choice to the more available animals, and therewith avoided elimination unlike the highly anthropophilic *An. gambiae* (*s.s*.) [40]. This adaptation to non-human hosts has also been observed in anthropophilic *An. gambiae* (*s.s*.) in Kenya [41]. Human biting rates for *An. funestus* were relatively similar indoors and outdoors. Comparable biting patterns have been reported in Uganda [42]. Changes in biting time preferences as well as increases in zoophily show how the host-seeking behaviour of *An. funestus* is dynamic and may change if pressured. Host-seeking activity was highest in the late hours of the night onto early morning hours. It has been reported that most rural families in the Kilombero Valley start their day around 5–6 am [8, 36] and so may become exposed to early morning biting vectors. Early morning feeding by *An. funestus* has also been reported in Benin [43], where up to 70 % of exposure to *An. funestus* bites occur after 6 am during daylight. In addition, in nearby villages insecticide susceptibility tests indicated high levels of resistance in *An. funestus* against deltamethrin (87 %), permethrin (65 %), lambda cyhalothrin (74 %), bendiocarb (65 %) and DDT (66 %). Similarly, *An. arabiensis* showed insecticide resistance to deltamethrin (64 %), permethrin (77 %) and lambda cyhalothrin (42 %) in 2014 [35], which may be associated with the reduced sensitivity of *An. funestus* to the pyrethroid transfluthrin. It is certainly necessary to understand this relationship before the consideration of a new pyrethroid-based spatial repellent into vector control.

The epidemiological significance of insecticide resistance on malaria transmission is unclear [44]. Ideally vector control should target the vector in multiple locations and physiological stages. Current strategies only control vectors indoors. Vectors are given sufficient room to maneuver their behavior outside the control zone. If elimination is to be reached vector control has to be played like a game of chess, where the opponent must be fought strategically with all available means targeting the vector at different stages of its life. Successes achieved through LLIN campaigns may be followed by a sharp increase in malaria transmission if strategies do not anticipate changes in the vectors behavior. It is likely that malaria will significantly increase in the Kilombero valley if the current situation is not re-evaluated and new tools implemented within the near future.

## Conclusions

A considerable degree of malaria transmission in the Kilombero Valley is occurring outside sleeping hours when people are unprotected. This gap in personal protection from disease vectors needs to be addressed. In areas where the main vector is *An. arabiensis*, transfluthrin spatial repellents may offer some complimentary control aimed at early evening outdoor biting vectors (23 % of exposure as measured in this study) that are precluded by LLINs since HLC demonstrated that the mosquito coils showed good efficacy in preventing mosquito landings. However, the data did not demonstrate a large decline in either mosquito density or HBI in the clusters of houses where the transfluthrin repellents were used despite showing a good efficacy in preventing mosquito landings as measured by human landing catches. Pyrethroid-based spatial repellents may provide a degree of personal protection; however the overall public health benefit is doubtful and potentially iniquitous as their use may divert malaria vectors to those who are not wealthy or wise enough to use them.

*An. funestus* proved to be insensitive to volatile pyrethroids, this is quite worrisome given its increasing presence in the region. The behavioural responses of susceptible and resistant *An. funestus* mosquitoes to pyrethroid volatiles needs to be rigorously characterised in order to understand if pyrethroid-based spatial repellents can play a role in malaria endemic areas where the main vectors are pyrethroid-resistant, or if there is an intrinsic species specific variability of response to transfluthrin. Successful implementation of integrated vector control strategies using volatile pyrethroid spatial repellents will be highly dependent on local vector composition, resistance to pyrethroids, feeding times and host preference. Entomological surveys must be made to areas where the implementation of spatial repellents is being considered in order to assess their suitability before implementation.

## Abbreviations

CI, Confidence interval; DEET, N,N-Diethyl-meta-toluamide; ELISA, enzyme linked immunosorbent assay; HBI, human blood index; HLC, human landing catch; IHI, Ifakara Health Institute; IHI-IRB, Ifakara Health Institute Institutional Review Board; IQR, inter-quartile range; IRR, incidence rate ratio; ITNs, insecticide-treated nets; LLINs, long-lasting insecticide-treated nets; mRDT, malaria rapid diagnostic test; NIMR, National Institute of Medical Research; OR, Odds ratio; PCR, Polymerase chain reaction; RR, Rate ratio
